# Epidemiological profile of acquired syphilis in children and adolescents in Brazil: a nationwide observational study

**DOI:** 10.3389/fped.2025.1670688

**Published:** 2026-01-29

**Authors:** Gabrielle Oliveira Silva, Amanda Souza Barbosa, Catarina David Rodamilans Ferreira, Igor Macedo Pinto, Bárbara Simone David Ferreira

**Affiliations:** 1 Center for Education and Research in Pediatrics (NEPP), Zarns Medical College, Salvador, Brazil; 2 Clariens Education, Zarns Medical College, Salvador, Brazil; 3 Medical Law Attorney, Federal University of Bahia Law School (UFBA), Salvador, Brazil; 4 Medicine Course, Salvador University (UNIFACS), Salvador, Brazil; 5 Afya Educational Group, UNIDOMPEDRO Medical School, Salvador, Brazil

**Keywords:** acquired syphilis, early diagnosis, epidemiological surveillance, nonsexual transmission, pediatrics, sexually transmitted infections

## Abstract

Acquired syphilis, which is historically considered a condition with almost exclusive prevalence in adults, has shown, in recent evidence, an alarming increase amongst the pediatric population. The objective of this study is to analyze and compare the time trends and epidemiological profile of acquired syphilis in the Brazilian population from ages 0 to 19 from 2015 to 2023. It consists of a descriptive, ecological study that utilizes secondary data from the Notifiable Health Conditions Information System (SINAN). The trend analysis used Joinpoint regression to calculate the Annual Percent Change (APC). From 2015 to 2023, 135,699 cases of acquired syphilis were registered in the age group studies, with 78.5% of confirmed cases and a concentration of 94.1% in the 15–19 age group. The results suggest a complex epidemiological scene, with the advancement of syphilis in various groups within the young population, evidencing the urgent necessity to strengthen prevention, diagnosis, and surveillance policies.

## 1 Introduction

The *Treponema* genus of bacteria contains both pathogenic and non-pathogenic species. *Treponema pallidum* is a pathogenic bacterium of global distribution that causes syphilis and that, for the past decades, has become a significant public health issue in several countries, particularly in developing ones, like Brazil (1). Human beings are the only source of treponemal infection, with no known non-human reservoirs. The highest rates of infection correspond to the most sexually active age groups (2).

Acquired syphilis (AS), an infection mainly sexually transmitted, differs from congenital syphilis, in which transmission occurs vertically, from an infected mother to her child during pregnancy or childbirth. AS can also occur through the transfusion of contaminated blood, the use of shared syringes, or accidental inoculation with sharp, contaminated instruments (3).

AS, which is historically considered a condition with almost exclusive prevalence in adults, has shown, in recent evidence, an alarming increase amongst the pediatric population, particularly in children and adolescents exposed to non-sexual transmission, in more complex investigations (4, 5). Syphilis in children, even though less common, can be a significantly challenging diagnosis, due to clinical variety and the possibility of being confused with other dermatoses or infectious diseases (6).

Studies conducted in different geographical regions revealed that non-sexual transmission, particularly through direct contact with infectious wounds in home environments, is a relevant form of transmission during childhood. Manuscripts evidence cases of children infected by everyday interactions with contaminated caregivers, highlighting the need to broaden the clinical and epidemiological approach beyond the assumption of sexual abuse (7).

Another study, by analyzing two and a half decades of records in a tertiary center in India, confirmed the continuous presence of the disease in pediatric patients, reinforcing the importance of active surveillance and health education, since AS in children did not receive the appropriate attention and, unfortunately, continues to be a relatively ignored area (8).

Syphilis is a curable infection known for centuries, which can present a long, progressive duration, with complex clinical manifestations, involving multiple systems and organs, and various forms of transmission (4). In the majority of cases, which occur via sexual transmission, syphilis starts with an ulcerous lesion in the anogenital region, and, as is the case with sexually transmitted infections, this type of lesion is very significant in the transmission of the human immunodeficiency virus (HIV) and hepatitis B and C (3).

Unfortunately, syphilis does not confer immunity, and reinfection as well as superinfection may occur (3). The outcomes associated with acquired syphilis in childhood can be severe, especially with late diagnosis. It has been observed that hospitalization for symptomatic forms of the infection is associated with significant complications, underscoring the importance of early screening strategies and timely treatment (9). In this context, the primary goal of this study was to raise and comprehend the epidemiological profile of acquired syphilis in Brazilian children and adolescents. Furthermore, to analyze the time trends of incidence rates of acquired syphilis in the same population from 2015 to 2023, contributing to the development of efficient public policies and the training of competent professionals to address this emerging condition.

## 2 Methods

### 2.1 Overall study design

This is a descriptive, time-series ecological study that used secondary data from the public domain. The notification data on acquired syphilis were extracted from the Information System for Notifiable Diseases (SINAN Net), whilst the population estimates from the Brazilian Institute of Geography and Statistics (IBGE).

The population studied was from ages 0–19, whose syphilis diagnoses were made and conducted in Brazil, regardless of the patient's nationality. Non-confirmed or with confirmation status unknown were excluded. The study describes and analyzes sociodemographic characteristics, distribution throughout the years, and the outcome on the child and adolescents in Brazil from ages 0–19 diagnosed with acquired syphilis.

### 2.2 Limitations

The syphilis cases in pregnant women in the age group studied are not available within the acquired syphilis dataset; they have a separate dataset that was not used in this study. It was decided not to incorporate the pregnant women dataset because it includes variables that do not align with those analyzed in this study.

Unfortunately, there is no data available regarding co-infection between *Treponema pallidum* and Acquired Immunodeficiency Syndrome virus. There is also no data on the route of transmission, despite its relevance, especially to distinguish between sexual transmission, which may involve abuse or sexual violence, and non-sexual transmission. Clinical manifestations are also unavailable. Therefore, this study does not address these points.

Other limitations of this study are a result of using secondary data, which includes the possibility of sub-notification, inadequate variable filling, and inconsistencies in record quality across different locations and time periods. These factors can influence the magnitude of the presented rates but not necessarily invalidate the observed trends that constitute the primary focus of this analysis.

### 2.3 SINAN, Brazilian ministry of health

The Brazilian Ministry of Health (MSB) implemented, maintains, and supports SINAN (10), a system that registers notifications and investigations of diseases classified as mandatory reporting by Brazilian legislation; amongst these diseases are congenital and acquired syphilis, as well as syphilis in pregnant women. When there is a suspicion or a confirmed case, it must be mandatorily reported in the system by a healthcare professional, through the completion of the SINAN Notification/Investigation Form, standardized for AS.

The collected data is then inserted into the DATASUS-SINAN system, which consolidates the information for local, regional, and national analysis, updating the epidemiological surveillance databases. SINAN provides the following information: date (day, week, month and year) of diagnosis and notification, region, geographic zone (despite not being included in the analysis of this study), state and city of residency and notification, age group, self-reported race/ethnicity, sex, with no reference to gender, educational level measured in years of schooling, diagnosis classified between blank or ignored, confirmed, descartes or inconclusive, evolution with the possibilities of left blank or unknown, cure, death due to the reported condition, death from another cause, death under investigation; and diagnostic criteria of left blank or unknown, clinical (without detailed reference to clinical data or manifestations), clinical-epidemiological (also without specific citation of clinical data), or laboratory (without detailing the tests performed).

Information such as syphilis antecedents, prior treatment, sexual behavior, clinical classification (Primary, Secondary, Tertiary, Latent), tests performed (treponemal and non-treponemal), and partner treatment is not available for AS; however, it is available for pregnant women, along with the gestational period at diagnosis.

The forms that update the system are of public access and can be consulted through SINAN's website:

http://portalsinan.saude.gov.br/images/documentos/Agravos/NINDIV/Notificacao_Individual_v5.pdf

http://portalsinan.saude.gov.br/images/documentos/Agravos/NINDIV/Ficha_conclusao_v5.pdf

As previously mentioned, AS is classified as a disease of mandatory reporting that can be reported based on initial suspicion. After the segment and conclusion of the investigation, the diagnosis is classified as confirmed or discarded according to the definitions of cases provided by the Health Surveillance Secretariat and the Infectious Diseases Guide of the Department of Epidemiological Surveillance (11), which the MSB also maintains.

The definitions of confirmed cases are the following:

(i) Every suspected case of syphilis (hard chancre; secondary mucocutaneous lesions (roseola, papular syphilids, condyloma lata, alopecia); tertiary mucocutaneous lesions [tubercles or gummas]; neurological alterations (tabes dorsalis, dementia); cardiovascular alterations (syphilitic aortitis, aortic aneurysm); articular alterations (Charcot's arthropathy) whose diagnosis is confirmed by laboratory tests, regardless of the presence of clinical signs and symptoms, including: reactive result in a treponemal test—such as FTA-Abs (Fluorescent Treponemal Antibody-Absorption), MHA-Tp (Microhemagglutination *Treponema pallidum* Assay), TPHA (*Treponema pallidum* Hemagglutination Assay), and ELISA (Enzyme-Linked Immunosorbent Assay); rapid tests for syphilis diagnosis (immunochromatographic tests); and reactive result in a non-treponemal test—VDRL (Venereal Disease Research Laboratory) or RPR (Rapid Plasma Reagin);
(ii) All suspected cases with a history of epidemiological link with a confirmed case (sexual partner with positive laboratory diagnosis), regardless of laboratory confirmation;

Moreover, the definitions of discarded cases include:

(i) present negative laboratory results (non-reactive treponemic and non-treponemic tests) and the absence of compatible clinical signs;
(ii) all suspected cases that have had a confirmed diagnosis of another disease;
(iii) cases with errors in the notification (ex: duplicity, incoherent data, absence of laboratory or epidemiological evidence).

### 2.4 Data analysis

Notifications of acquired syphilis were analyzed for the age groups <1 year, 1–4, 5–9, 10–14, and 15–19 years, from January 2015 to December 2023. The data were analysed in descriptive form, presented in absolute (n) and relative (%) frequencies.

For the trend analysis, the Joinpoint Regression Program was used. This method identifies inflection points where statistically significant changes occur in the trend and calculates the Annual Percent Change (APC) for each segment. A trend was considered statistically significant when the 95% confidence interval (95% CI) did not include zero. The following rates were calculated for AS (0–19 years): incidence rate per 100,000 inhabitants, stratified by age groups (0–4, 5–9, 10–14, and 15–19 years).

### 2.5 Ethical aspects

All the data presented in this study have been provided by SINAN, through DATASUS, a platform maintained by the Brazilian Ministry of Health. This study was conducted in accordance with Resolution No. 466/12, which regulates research involving human beings in Brazil. All the information was anonymized and made publicly available, and the study did not require consent for participation or ethical approval.

## 3 Results

Between January 2015 and December 2023, a total of 1,404,815 cases of AS were notified and registered in the SINAN-acquired syphilis database, with 135,699 of the AS notifications from the studied age group (9.65%). All the notified cases were analyzed.

### 3.1 Sex

**Figure 1:**
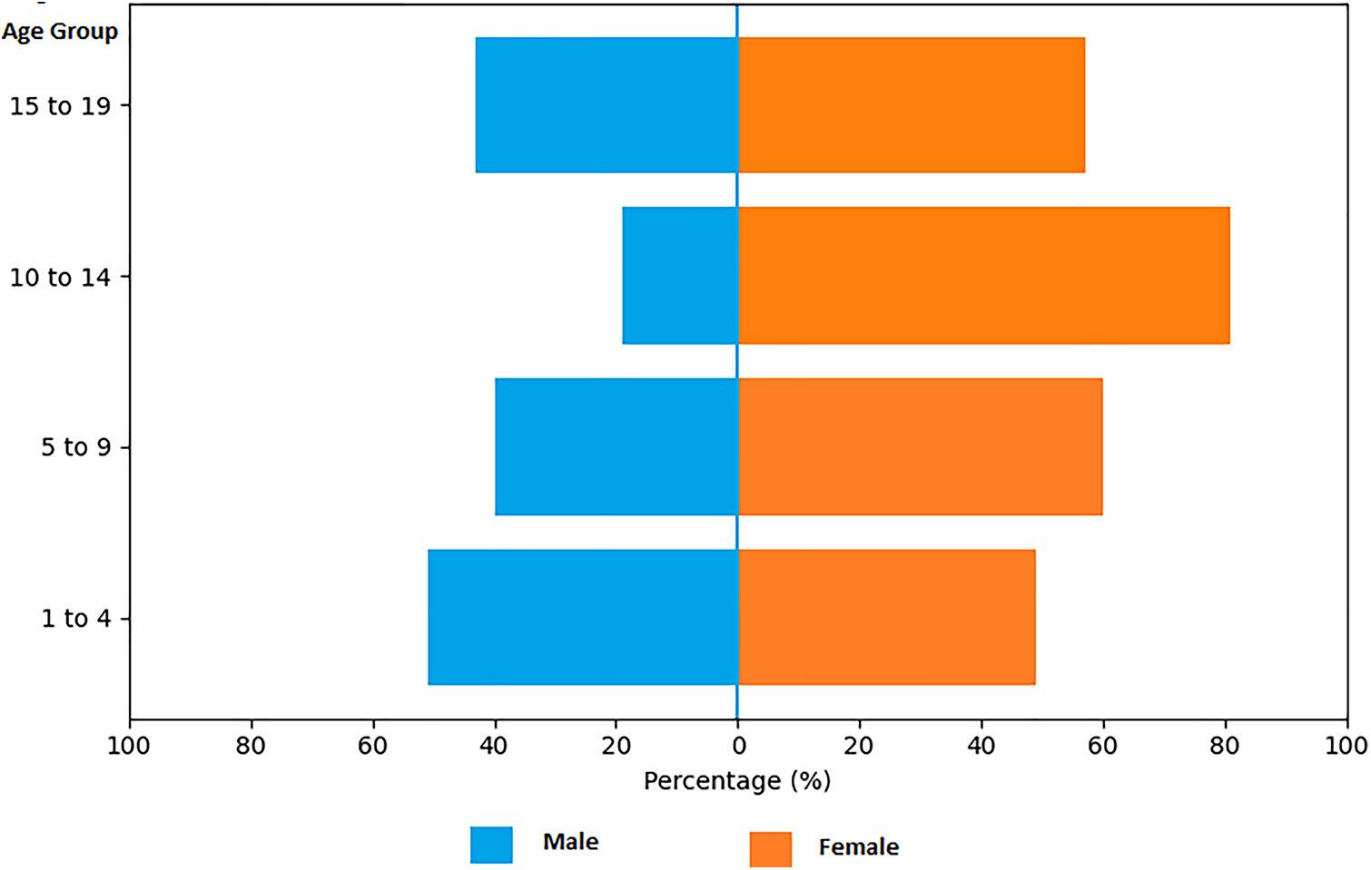
Sex percentage chart separated by age group, Brazil, 2015–2023. Source: Prepared by the authors (2025), based on Ministry of Health/SVS – Information System for Notifiable Diseases (SINAN Net).

The sex distribution showed a slight predominance of females (52.1%), followed by males (42.7%), and unknown, which accounted for less than 0.1% of the cases, as shown by age group in Figure 1. Despite this distribution, there was no statistically significant difference between the sexes (chi-square test; *p* = 0.08). There is no gender distinction in the database.

When analyzing each age group separately, there was no statistically significant difference amongst sexes in patients aged 1–4, however, in the other age groups (5–9, 10–14, and 15–19), there was a significant prevalence in female patients compared to male (*p* < 0.05).

### 3.2 Ethnicity

**Figure 2:**
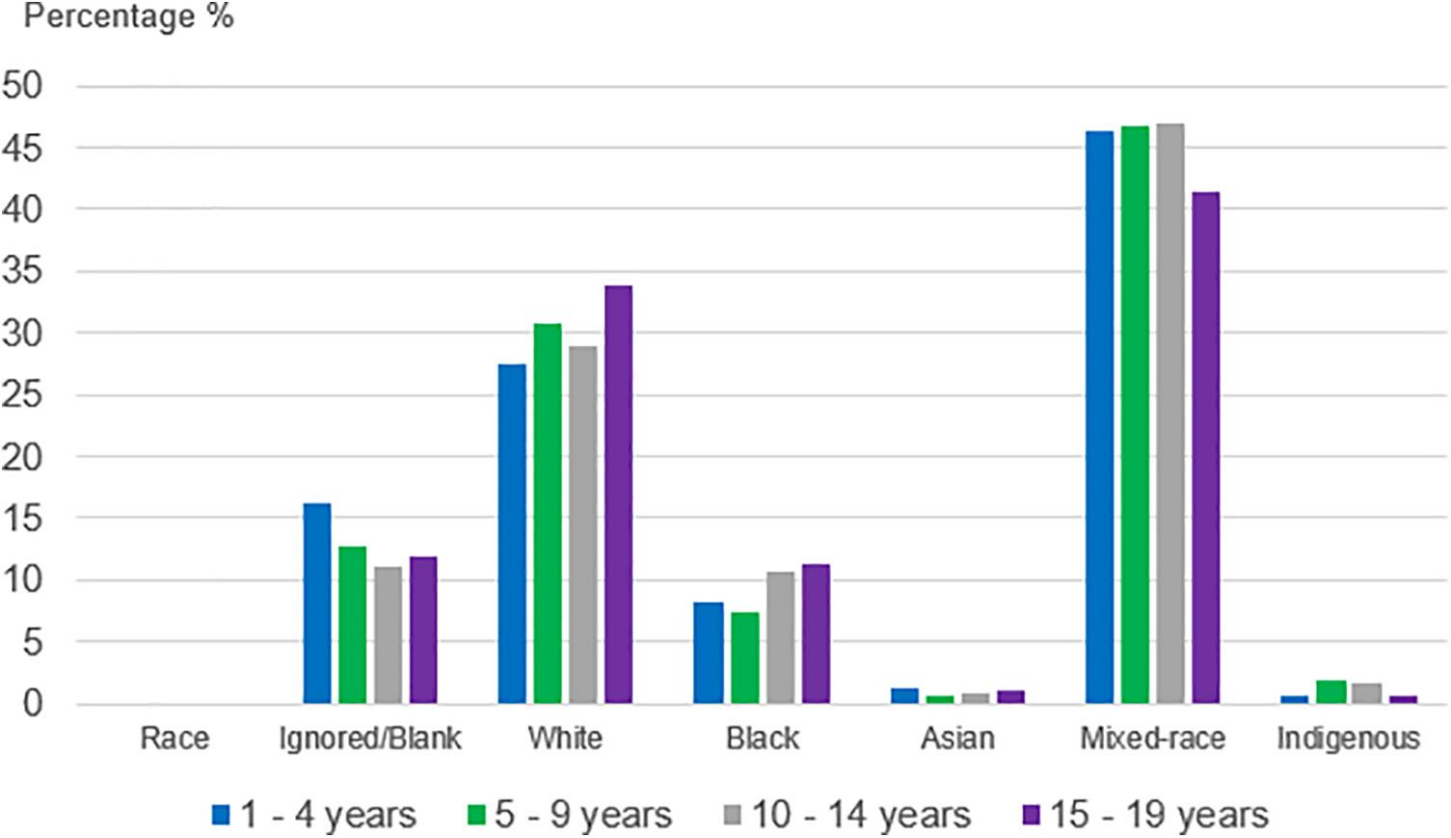
Distribution of acquired syphilis cases in Brazil (2015–2023), divided by ethnicity per age group. Source: Prepared by the authors (2025), based on Ministry of Health/SVS – Information System for Notifiable Diseases (SINAN Net).

The majority of individuals self-reported mixed race (41.6%), followed by white (33.5%), unknown/blank (12%), black (11.1%), Asian (1%), and Indigenous (0.7%). The ethnic distribution per age group is shown in Figure 2. The findings are consistent with the Brazilian demographic profile.

### 3.3 Educational level

Regarding educational levels, the majority of patients had between 5 and 11 years of study (57%), which is consistent with the expected for adolescents, the most affected age group, with only 2.5% classified as illiterate, while 0.4% of the sample does not fit in education level categories, since they are children under 5 years of age. The lowest educational levels were in the Northeast, with 23 cases per 100,000 inhabitants in the most affected age group, reaching those with 5–11 years of schooling, followed by the North (40.2/100,000), while the best rates were found in the South (80.3/100,000), followed by the Southeast (67.2/100,000).

### 3.4 Case classification and confirmation criteria

The majority (78.5%) was classified as confirmed, followed by inconclusive (18%). Only 0.7% were discarded.

Among the confirmed cases, the predominant criterion for confirmation was laboratory testing (99.2%), as shown in Table 1.

**Table 1:** Distribution of acquired syphilis notifications by classification and confirmation criteria, Brazil, 2015–2023.

Classification	Ignored/Blank (*n*/%)	Laboratory (*n*/%)	Clinical-epidemiological (*n*/%)	Total (*n*/%)
Ignored/Blank	3,826 (12.7%)	1 (0.0%)	0 (0.0%)	3,827 (2.8%)
Confirmed	1,920 (6.4%)	94,157 (99.2%)	10,431 (96.8%)	106,508 (78.5%)
Discarded	106 (0.4%)	616 (0.6%)	278 (2.6%)	1,000 (0.7%)
Inconclusive	24,166 (80.5%)	131 (0.1%)	67 (0.6%)	24,364 (18.0%)
Total	30,018	94,905	10,776	135,699

Prepared by the authors (2025), based on Ministry of Health/SVS – Information System for Notifiable Diseases (SINAN Net).

### 3.5 Distribution by age group and outcome

The age group analysis (Table 2) reveals a striking concentration of cases (94%) in the 15- to 19-year-old age group. Regarding the outcomes, most cases in all age groups resulted in a cure, despite the number of unknown or blank being significant.

**Table 2:** Distribution of acquired syphilis cases by age group and case outcome. Brazil, 2015–2023.

Age group	Unknown/Blank (*n*/%)	Cure (*n*/%)	Death due to condition (*n*/%)	Death from other cause (*n*/%)	Total (*n*/%)
0–4 years	322 (0.5%)	230 (0.33%)	1 (1.54%)	0 (0.0%)	553 (0.41%)
5–9 years	279 (0.4%)	268 (0.38%)	0 (0.00%)	0 (0.0%)	547 (0.40%)
10–14 years	3,362 (5.1%)	3,574 (5.09%)	6 (9.23%)	1 (2.3%)	6,943 (5.12%)
15–19 years	61,359 (93.9%)	66,196 (94.21%)	58 (89.23%)	43 (97.7%)	127,656 (94.07%)
Total	65,322	70,268	65	44	135,699

Prepared by the authors (2025), based on Ministry of Health/SVS – Information System for Notifiable Diseases (SINAN Net).

Pediatric groups had higher prevalence of unknown or blank data, 58% and 51%, for ages 0–4 and 5–9 years-old, respectively, while both adolescent groups had 48% each.

### 3.6 Geographic and temporal distribution of confirmed cases

**Figure 3:**
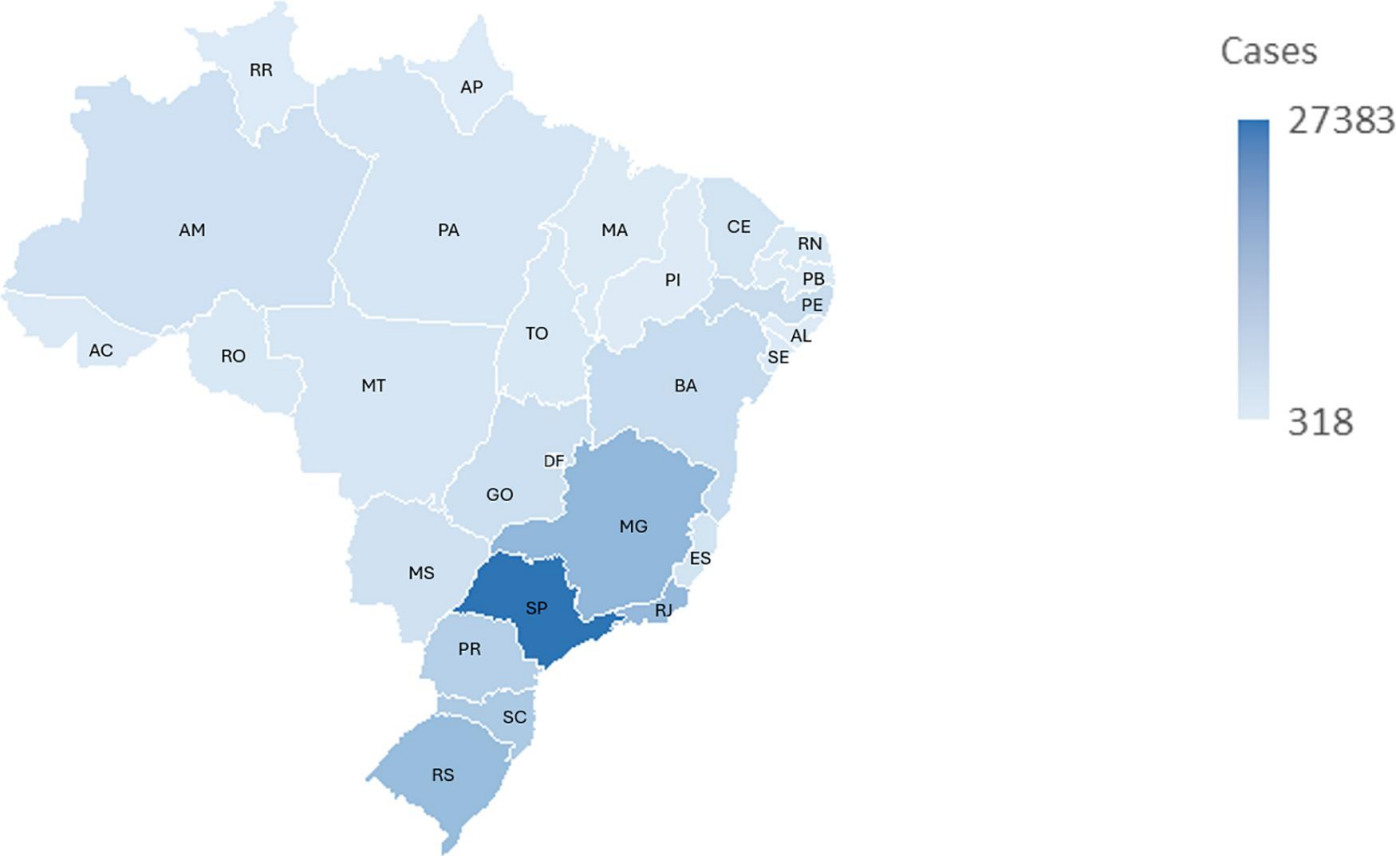
Confirmed cases aged 0–19 years, by federative unit (UF) of residence and year of notification. Brazil, 2015–2023. Source: Prepared by the authors (2025), based on Ministry of Health/SVS – Information System for Notifiable Diseases (SINAN Net).

 Figure 3 shows the distribution of confirmed cases by Federative Unit (UF) of residence over the years. A progressive increase in the number of notifications can be observed in most states, with the largest absolute numbers concentrated in the most populous states of the Southeast region (São Paulo - SP, Minas Gerais - MG, and Rio de Janeiro - RJ) and the South region (Rio Grande do Sul - RS). Espírito Santo - ES, a state in the Southeastern region, has not provided updated data in recent years.

**Figure 4:**
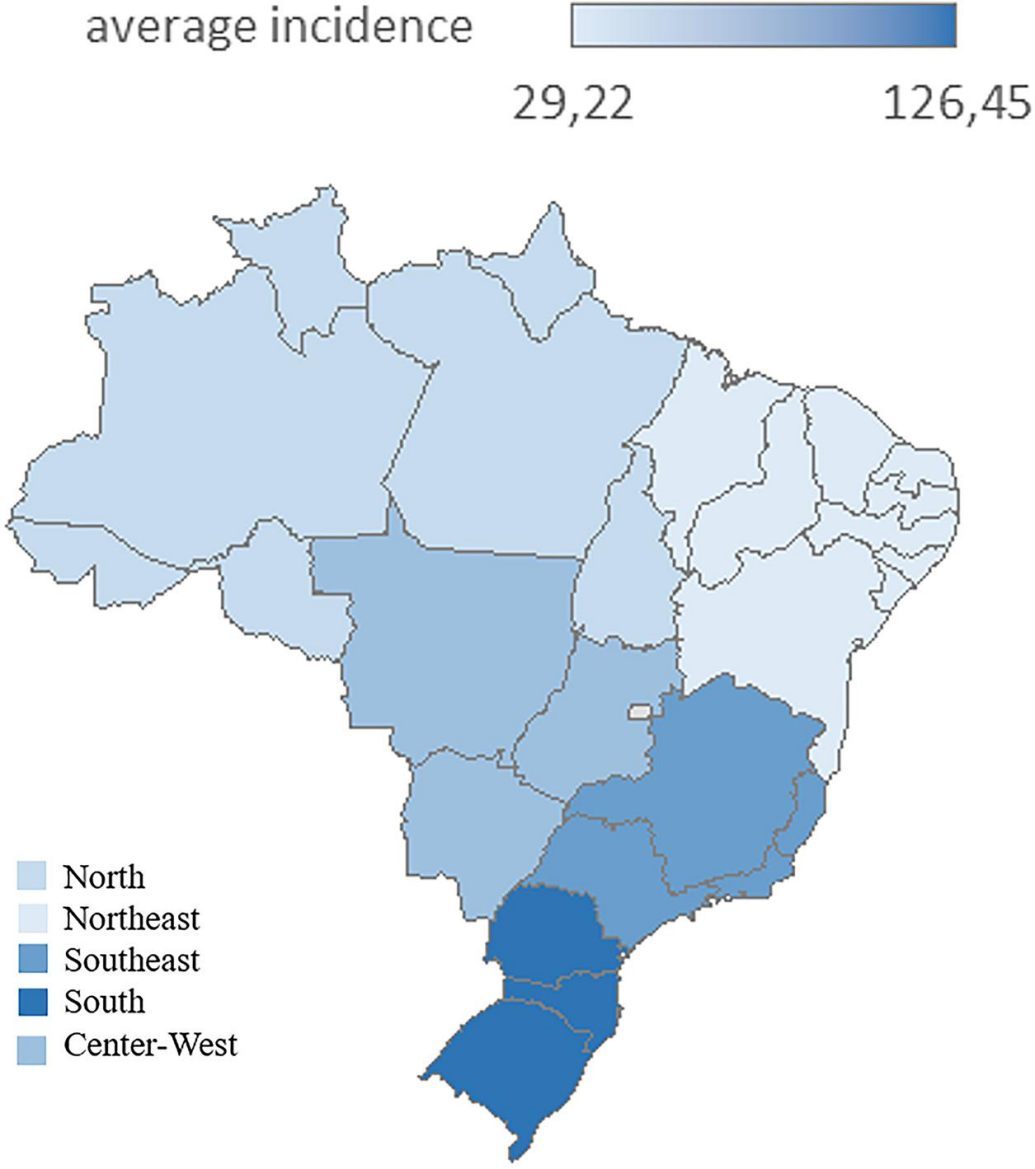
Acquired syphilis average incidence per 100,000 inhabitants by region of residence, all age groups, Brazil, 2015–2023. Source: Prepared by the authors (2025), based on Ministry of Health/SVS – Information System for Notifiable Diseases (SINAN Net).

Tables 3–6 display the incidence rates (per 100,000 inhabitants) per region, stratified by age group, while Figure 4 shows the incidence per 100,000 group by region for all age groups. The analysis evidences the following:

(a) In the younger age groups (ages <1 to 5–9), the incidence rates are very low and unstable
(b) In the 10–14 age group, the rates have started to increase, especially in the Southeast and Midwest, in recent years
(c) In the 15–19 age group, the rates are substantially higher, with the South and Southeast consistently presenting the higher incidences, exceeding 180 cases per 100.000 inhabitants in the South in 2023.

**Table 3:** Acquired syphilis rate per 100,000 inhabitants by region of residence, age groups <1 year and 1–4 years, Brazil, 2015–2023.

Region of residence	2015	2016	2017	2018	2019	2020	2021	2022	2023
North	0.06	0.19	0.12	0.25	0.31	0.00	0.19	0.31	0.68
Northeast	0.05	0.05	0.07	0.14	0.24	0.00	0.17	0.39	0.48
Southeast	0.07	0.07	0.35	0.38	0.26	0.00	0.38	0.48	0.80
South	0.16	0.42	0.21	0.56	0.20	0.05	0.20	0.45	0.96
Center-West	0.00	0.00	0.17	0.25	0.17	0.00	0.08	0.49	1.31

Prepared by the authors (2025), based on Ministry of Health/SVS – Information System for Notifiable Diseases (SINAN Net).

**Table 4:** Acquired syphilis rate per 100,000 inhabitants by region of residence, age group 5–9 years, Brazil, 2015–2023.

Region of Residence	2015	2016	2017	2018	2019	2020	2021	2022	2023
North	0.06	0.18	0.24	0.36	0.49	0.00	0.25	0.37	0.12
Northeast	0.02	0.04	0.29	0.32	0.16	0.00	0.24	0.19	0.36
Southeast	0.19	0.21	0.43	0.43	0.43	0.04	0.35	0.40	0.64
South	0.47	0.47	0.37	0.54	0.65	0.00	0.37	0.83	0.61
Center-West	0.09	0.00	0.17	0.35	0.17	0.00	0.26	0.42	0.41

Prepared by the authors (2025), based on Ministry of Health/SVS – Information System for Notifiable Diseases (SINAN Net).

**Table 5:** Acquired syphilis rate per 100,000 inhabitants by region of residence, age group 10–14 years, Brazil, 2015–2023.

Region of Residence	2015	2016	2017	2018	2019	2020	2021	2022	2023
North	0.57	1.73	1.98	3.63	3.05	2.18	2.89	3.99	6.87
Northeast	0.56	1.12	1.62	2.53	1.90	1.23	2.29	2.56	3.64
Southeast	1.99	3.13	4.07	5.31	5.82	4.57	5.17	7.07	7.71
South	0.56	0.86	1.47	2.71	2.87	1.56	2.11	3.47	4.42
Center-West	0.98	1.48	2.48	4.47	4.67	2.52	3.38	5.54	7.04

Prepared by the authors (2025), based on Ministry of Health/SVS – Information System for Notifiable Diseases (SINAN Net).

**Table 6:** Acquired syphilis rate per 100,000 inhabitants by region of residence, age group 15–19 years, Brazil, 2015–2023.

Region of residence	2015	2016	2017	2018	2019	2020	2021	2022	2023
North	7.30	14.75	22.09	36.48	43.53	33.63	54.45	66.68	72.94
Northeast	8.49	12.99	21.78	33.08	32.14	20.01	31.76	36.86	45.29
Southeast	38.03	51.33	75.06	87.00	102.32	78.55	97.79	119.75	136.94
South	47.64	81.83	109.17	141.25	138.05	112.52	131.99	167.52	180.57
Center-West	15.88	22.15	41.24	70.12	64.19	51.65	66.34	87.08	127.92

Prepared by the authors (2025), based on Ministry of Health/SVS – Information System for Notifiable Diseases (SINAN Net).

Regarding the time trend of AS in children and adolescents (0–19 years of age), the analysis identified a distinct trend between the different age groups for acquired syphilis. Table 7 summarizes the results of the Joinpoint regression.

**Table 7:** Annual percentage change (APC) in the incidence rate of acquired syphilis, by age group. Brazil, 2015–2023.

Age group	Time Period	APC (%)	IC 95%
0–4 years	2015–2017	0.0	0.0–0.0
	2017–2023	0.0	0.0–0.0
5- 9 years	2015–2017	0.0	0.0–0.0
	2017–2023	0.0	0.0–0.0
10–14 years	2015–2023	12.8	3.6–25.1
15–19 years	2015–2017	54.3	18.7–115.5
	2017–2023	9.3	−6.5–15.3

Prepared by the authors (2025), based on data from Joinpoint Regression software.

**Figure 5:**
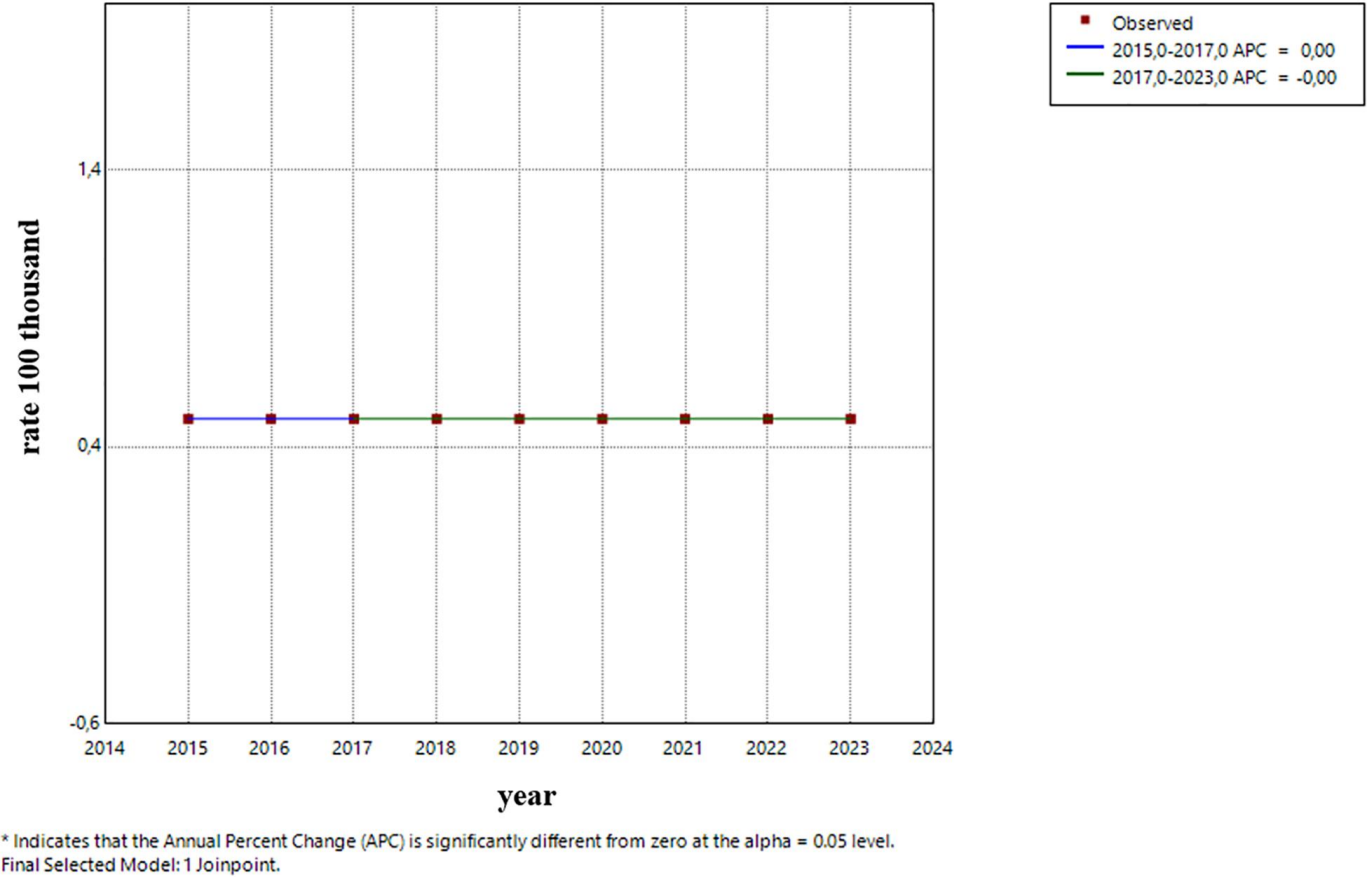
Trend (Age group 0–4 years).

**Figure 6:**
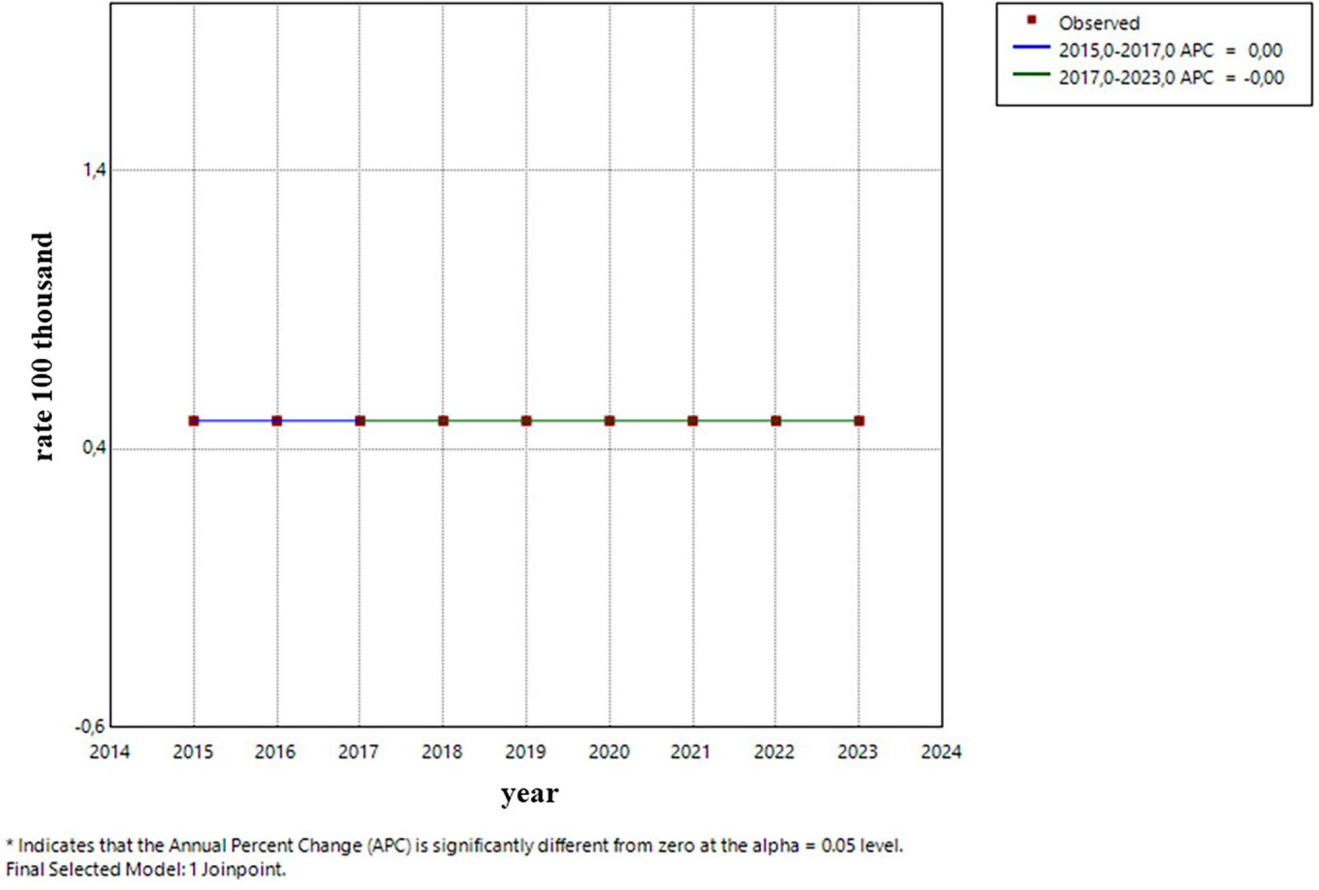
Trend (Age group 5–9 years).

**Figure 7:**
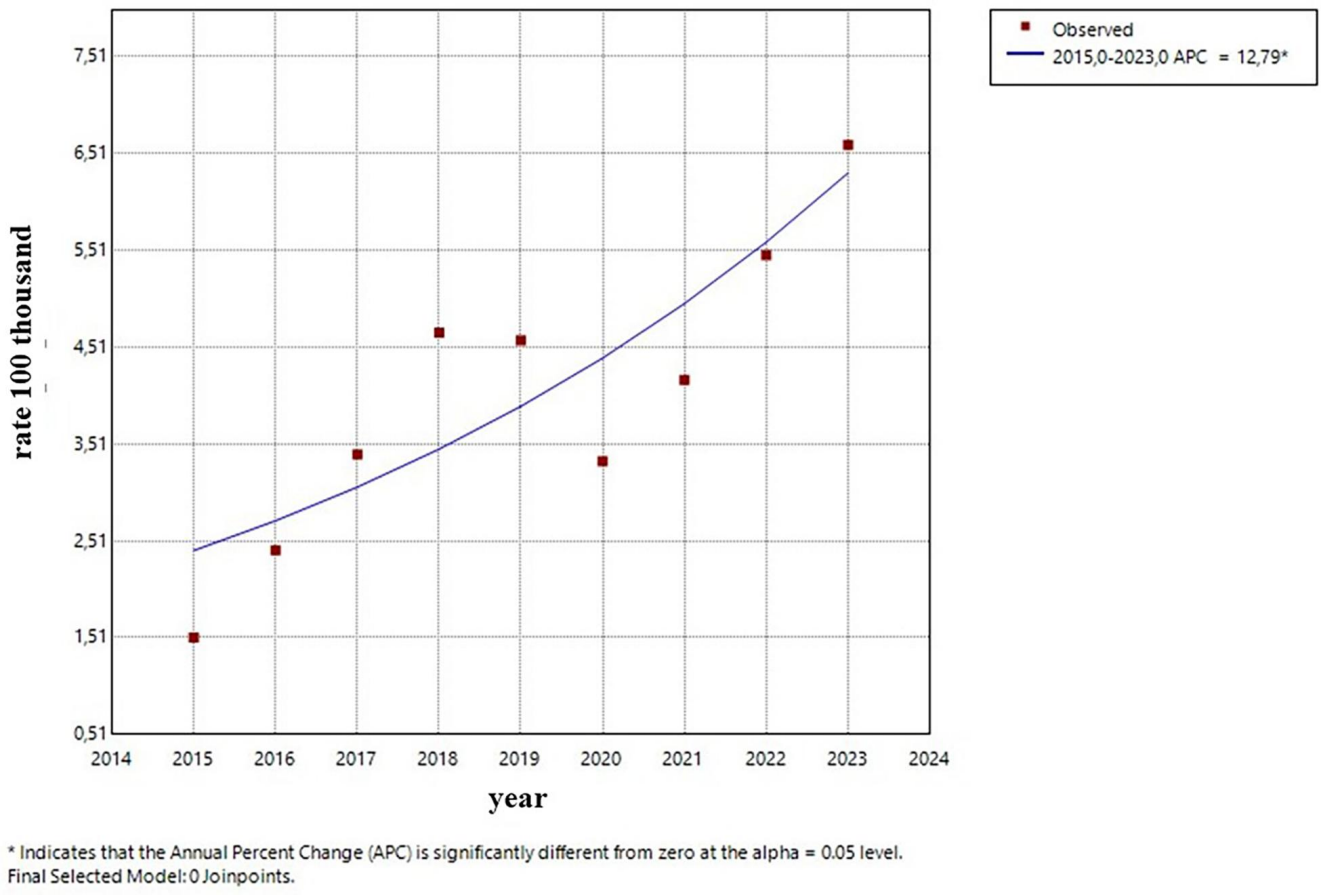
Trend (Age group 10–14 years).

**Figure 8:**
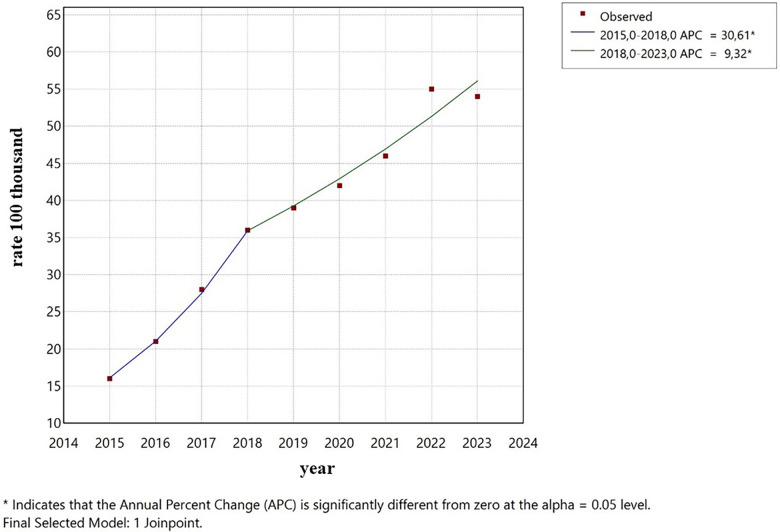
Trend (Age group 15–19 years).

Trend patterns:

- 0–4 years old: After a period of stability, the incidence rate showed an increase starting in 2021, with no significance. (Figure 5).
- 5–9 years old: The trend remained stable during the entire period (Figure 6).
- 10–14 years old: A constant and significant rise was observed throughout the entire time period (Figure 7).
- 15–19 years old: The group showed an exponential growth until 2017, followed by a deceleration (Figure 8).

### 3.7 Data not publicly available or inconsistent

The public data does not allow the analysis of the route of transmission and co-infection with HIV, despite the importance of investigating co-infection between different sexually transmitted infections (12). In the case of acquired syphilis, the state of the disease, the clinical manifestations, the record of the diagnostic tests performed, and whether there was a previous diagnosis of the disease are not publicly available.

Although it was possible to quantify the cases in females, it was not possible to determine precisely the presence or absence of a simultaneous pregnancy at the time of diagnosis in the notifications. This limitation is due to the incompatibility in the registered data in the SINAN - pregnant syphilis with the SINAN - acquired syphilis databases. This inconsistency compromises the trustworthiness of the information related to the gestational condition of the patients at the moment of the notification.

## 4 Discussion

Despite being a pathology known for centuries, syphilis is a great problem in global public health. According to the Sexually Transmitted Diseases Bulletin of the United States Centers for Disease Control and Prevention (CDC), the incidence of primary and secondary syphilis has been growing since 2001 (13).

Worldwide data suggests a rise in the number of cases in recent years, due to various factors, which can mainly impact the youth. These factors include changes in sexual behavior and a decrease in the fear of being infected by HIV, due to the use of HIV Pre-Exposure Prophylaxis (PrEP), even though it still lets them exposed to other STIs (3). The need for vigilance of the acquired syphilis made its notification mandatory in 2010 in Brazil (14).

In 2017, following recommendations from the Pan American Health Organization, the Brazilian Ministry of Health updated the definitions of syphilis cases, broadening notifications. Regarding acquired syphilis, for epidemiological surveillance purposes, two case-defining criteria were established, referred to as Situation 1 and Situation 2, as shown in Table 8 (15).

**Table 8:** Definition of case notification of acquired syphilis.

Situation 1	Situation 2
Asymptomatic individual, with a reactive non-treponemal test at any titer and a reactive treponemal test, with no record of previous treatment.	Individual symptomatic for syphilis, with at least one reactive test - treponemal or non-treponemal - with any titer.

Prepared by the authors (2025), based on data from Health Surveillance Guide - Brazil.

These definitions enable the inclusion of individuals with syphilis at any clinical stage of the disease, especially when classified under Situation 1. However, this change had a minimal impact on case numbers in the studied population, unlike the adult population, which showed a substantial increase in case numbers at SINAN. This indicates that, even though these changes have a potential impact on diagnosis, they have not reached the pediatric and adolescent population yet.

In this study, the epidemiological characteristics publicly available in SINAN-acquired syphilis database were investigated, from 2015 to 2023, in Brazil, in patients aged 0–19, diagnosed with AS. More specifically, differences across age groups, ethnicities, and sexes, as well as mortality rates, were explored.

As previously mentioned, the prevalence of the disease is significant in Brazil, and over 9% of acquired syphilis cases affected children and adolescents; however, this theme is little explored nationally and internationally. By revising the literature available in the NCBI database in the last 10 years, it is possible to identify that “children” AND “acquired syphilis” have limited references.

Even though we can not affirm based on our dataset since it lacks information about the route of transmission, existing literature suggests that most acquired syphilis cases in children result from sexual abuse. It is essential to remember that it is a population that is potentially exposed to different forms of violence, of psychological, physical, and sexual nature, in addition to neglect or abandonment.

The Atlas of Violence 2025 (16) reveals the rise of cases of violence and neglect or abandonment in all age groups from 0 to 19, especially in the family environment, in recent years.

Data from IPEA (16) indicates the type of violence more prevalent in each age group: infants are more often victimized by neglect, children by psychological and sexual violence, and adolescents by physical violence. With that being said, the tendency is to consider that contaminated infants are more likely to be caused by neglect in caregiving from their families, especially when it comes to hygiene and the failure to adopt barrier methods to prevent contact of the child with active skin lesions, and older children by sexual violence.

For this study, it is worth clarifying that neglect refers to the failure to provide the basic needs and care required for the full development of a child or adolescent, such as inadequate healthcare and poor hygiene, with abandonment representing its most extreme form (16). And, as previously mentioned, according to Brazilian law, practicing any form of sexual act with a person under the age of 14 is a crime, namely statutory rape. Thus, there is no reason to discuss the possibility of an early onset of sexual activity in this age group (17).

Comparing ecological data to the data regarding the exposure of children and adolescents to multiple forms of violence allows the development of hypotheses for future research, considering the comprehension of the social determinants surrounding acquired syphilis in this group.

It is difficult to evaluate the incidence of childhood syphilis resulting from close family contact, from non-sexual origin. Additionally, due to the severity and frequency, the possibility of acquired syphilis originating from sexual abuse should always be eliminated. Acquired syphilis in children is not only a medical problem, but also a social problem that has been neglected for a long time.

Still, other routes can be considered, such as kisses, fomites, pre-chewed food, or in everyday care, where direct contact may occur between the child's mucosa or injured skin and the caregiver's open syphilitic lesions, reinforcing the need for strict hygiene measures and the use of protective barriers in both clinical and household settings (4).

Syphilis is not transmitted through breastmilk; however, a mother with syphilitic lesions in the skin should be more cautious during breastfeeding. When restricting research in the literature to *T. pallidum* contamination via non-sexual routes, there is less availability, as reported by Yang WJ et al. (4), who reviewed 30 years of literature in the same database while presenting a case report.

Coming across acquired syphilis cases in patients aged 1–4 (0.41%) in Brazil is not among expectations, considering that the majority of cases in the literature are linked to an active sex life (3, 5, 8). The elevated incidence of cases in adolescents in the studied population (99.19%), however, is consistent with the findings in the literature and with the beginning of the sex life. Even so, there are two age ranges of adolescents to consider, 10–14 years and 15–19 years.

Nevertheless, the analysis of the trend in acquired syphilis in the pediatric and adolescent population in Brazil reveals a complex and alarming scenario.

The stability observed in the 0–9 age group can seem, at first glance, a positive indicator. However, the absence of growth in the trend does not minimize the severity of each case. It is essential to highlight that studies have shown that the presence of sexually transmitted infections (STIs) in children can be an important clinical indicator of sexual violence; thus, it should be rigorously investigated by multidisciplinary healthcare teams and child protection (18).

The continuous and statistically significant growth in the 10–14 age group (APC of +12.8%) is particularly alarming. Thus, public policies have to be more precise regarding sex education and STI prevention. The Brazilian legislation considers any sexual act in people under the age of 14 to be statutory rape, which gives these data a dimension not only of public health, but also of child and adolescent safety and protection (19).

In the 15–19 age group, the considerable growth of +54.3% a year from 2015 to 2017 reflects the intensification of the syphilis epidemics in Brazil, documented in several epidemiological reports of the Ministry of Health. This increase can probably be attributed to a combination of factors, including a decline in condom use, increased testing, and enhancements to the notification system (18). The subsequent deceleration in the trend, beginning in 2017, although not significant, may indicate stabilization in the epidemics at high levels or the result of health policies implemented during that period. However, the incidence rates continue to be high, establishing adolescents and the youth as a key population for the control of the disease.

It is important to remember that, due to the screening policies in susceptible populations, congenital syphilis caused by vertical transmission (from mother to fetus) is typically diagnosed in the neonatal period or in the early years of life, having less chances of diagnosis in older children or adolescents.

Regarding the classification of syphilis, it can be made based on the time of progression of the disease: early, up to one year after the infection, and late, after one year; or through stages of evolution (primary, secondary, latent, and tertiary) (3).

Studies show that in sexually abused children, the syphilitic chancre usually occurs in the perineum; it is typically a painless (4), non-pruritic lesion that indicates the site of bacterial inoculation in the body, appearing on average 21 days after infection and evolving into an erosion (3). Since small children and babies obviously do not share information about their condition, physical alterations can be difficult to diagnose and treat in a timely manner. Older children can also have difficulty identifying and describing their lesions to caregivers, as they may not notice the lesions that are visible to them.

This difficulty in identifying the lesions constantly causes primary syphilis cases to evolve into secondary syphilis. Active lesions, particularly during the primary and secondary stages of the disease, are active transmission routes; thus, these patients can potentially disseminate the infection. Another point to consider is that, even when identified, syphilis cutaneous manifestations are frequently mistaken with other conditions, such as aphthae, tonsillitis, psoriasis, cutaneous leishmaniasis, and cat scratch disease (4).

Most people with syphilis are asymptomatic, which contributes to maintaining the chain of transmission, which is higher in the early stages (primary and secondary syphilis) of the infection, gradually decreasing over time (15). One of the changes expected with the implementation of PCDT is an increase in diagnoses among asymptomatic patients.

Unfortunately, as previously mentioned, there is no public data available regarding the stage or clinical manifestations of the disease. Since syphilis is one of the great mimickers in medicine, it would be interesting to evaluate its signs and symptoms; however, in addition to not having such data available in our case series, no references have been found in our search in NCBI/PubMed and Lilacs regarding the percentage of typical and atypical clinical manifestations of acquired syphilis in children and adolescents in the past 10 years.

In the analyzed data set, it has been found a difference in the number of cases in the different regions of the country, which indicates that despite absolute population factors influence the volume of notifications, the proportional burden of the disease is more pronounced in areas with a smaller young population contingent, like the South, suggesting the need for different regional strategies to intensify prevention, diagnosis and early intervention, as cited in literature when it comes to vulnerable populations (20–22).

The database is related to the notification forms and, therefore, to their content and how they are filled out by the attending physicians, potentially generating significant information losses. Additionally, it is also operator-dependent; that is, the speed with which the system is updated and the verification of data transcription can cause delays in accurate updates, which led us not to include the year 2024 and the first half of 2025.

## 5 Conclusion

By analyzing acquired syphilis in the Brazilian pediatric population, based on data provided by SINAN from 2015 to 2023, an alarming scenario that requires attention from healthcare authorities and society has been revealed. Even though the majority of cases are among adolescents from ages 15–19, which was already expected based on previous studies, due to its association to sexual activity, the presence of acquired syphilis in children with less than 10 years of age, particularly in the 0–4 age group, raises an important question about the possibility of transmission caused by neglect of sexual violence.

The unequal distribution among the different Brazilian regions and the highest proportional incidence in areas with a smaller youth population underscore the necessity for regionalized coping strategies, indicating that although it is a national problem, it must be addressed in a targeted manner for each region.

As previously mentioned, the limitations of this study are due to the use of secondary data, with the possibility of sub-notification and the absence of additional variables for a more in-depth analysis. Nevertheless, the robustness of the method and the nationwide coverage of the data confer validity to the results.

Thus, due to the elevated presence of the disease and its possible relation with vulnerability, neglect, and violence, it is fundamental that healthcare professionals are prepared for early diagnosis, a sensitive approach, and appropriate referral, contributing to the prevention of severe outcomes and the promotion of comprehensive health for this population.

## Data Availability

The original contributions presented in the study are included in the article/Supplementary Material, further inquiries can be directed to the corresponding author.
